# Nanofiber-Based Innovations in Energy Storage Systems

**DOI:** 10.3390/polym17111456

**Published:** 2025-05-23

**Authors:** Iva Rezić Meštrović, Maja Somogyi Škoc

**Affiliations:** 1 Department of Applied Chemistry, Faculty of Textile Technology, University of Zagreb, 10000 Zagreb, Croatia; 2 Department of Materials, Fibers and Textile Testing, Faculty of Textile Technology, University of Zagreb, 10000 Zagreb, Croatia; maja.somogyi@ttf.unizg.hr

**Keywords:** energy storage, sustainable solutions, net zero energy, genetically modified fibers, fibers for energy storage

## Abstract

Nanofibers have emerged as transformative materials in the field of energy storage, offering unique physicochemical properties such as high surface area, porosity, and tunable morphology. Recent advancements have also introduced genetically modified fibers—engineered at the biological level to produce functionalized nanostructures with customizable properties. These bioengineered nanofibers add a sustainable and potentially self-healing component to energy storage materials. This paper reviews key applications of conventional and genetically modified nanofibers in lithium-ion and sodium-ion batteries, supercapacitors, hybrid systems, and flexible energy storage with a focus on how genetic and molecular engineering of fibrous materials enables new capabilities in ion transport, electrode architecture, and device longevity. Together, these advances contribute to the development of next-generation energy storage systems with enhanced performance, biocompatibility, and sustainability. This review therefore critically examines the current state, advantages, and limitations of both synthetic and biopolymer-based materials in energy storage applications. It discusses recent technological innovations, such as polymer–nanoparticle composites, functionalized polymer matrices, and next-generation polymer electrolytes. Future research should prioritize enhancing conductivity, improving scalability, and reducing environmental impact, ensuring that polymer-based materials contribute to the development of more efficient and sustainable energy storage technologies.

## 1. Introduction

The development of advanced energy storage technologies is increasingly reliant on innovative materials that combine high performance with environmental sustainability. Among these, polymers have emerged as versatile components in batteries, supercapacitors, and fuel cells, owing to their lightweight nature, mechanical flexibility, and chemical tunability. Both synthetic and biopolymer-based materials are actively investigated for use as electrolytes, separators, and electrode constituents. Notably, conductive polymers such as polyaniline (PANI) and polypyrrole (PPy) offer promising redox activity, while polymeric electrolytes like polyethylene oxide (PEO) and polyvinylidene fluoride (PVDF) exhibit favorable ionic conductivity. Despite these advantages, conventional synthetic polymers often raise environmental concerns due to their persistence and toxicity, prompting a shift toward sustainable alternatives derived from renewable sources.

Biopolymers such as nanocellulose, lignin, and chitosan are thus gaining traction for their biodegradability and inherent functionality. Several intrinsic limitations—such as low electrical conductivity, limited thermal and chemical stability, and suboptimal ionic transport—continue to hinder the full realization of polymer-based energy storage systems. To overcome these challenges, recent research has explored the design of hybrid polymer composites, solid-state polymer electrolytes, and self-healing materials. Of particular interest is the development of biopolymer-derived carbon electrodes and biodegradable polymer membranes, which offer promising pathways toward green and high-efficiency storage devices.

**Figure 1:**
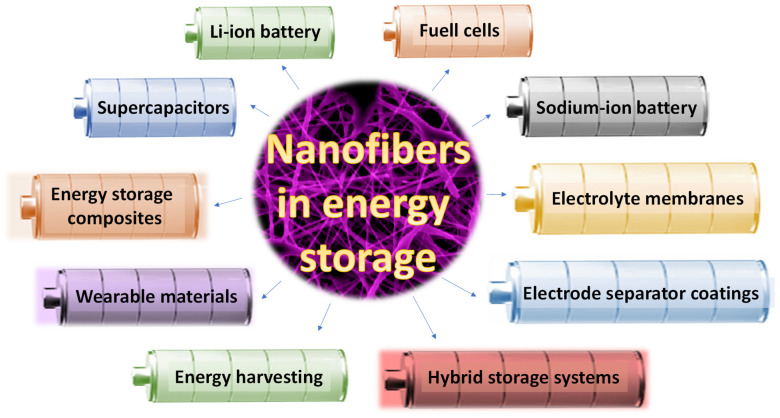
Graphical presentation of the nanofiber-based innovations in energy storage.

In parallel, nanofiber-based materials have garnered substantial attention due to their high surface area, tunable architecture, and mechanical robustness, all of which are critical for enhancing charge storage and transport. Advances in synthetic biology have further expanded the functional landscape of nanofibers by enabling the production of genetically engineered fibers with bespoke properties. These include enhanced conductivity, selective ion-binding capabilities, and improved structural integrity—attributes that are increasingly vital for next-generation energy storage technologies. This review provides a comprehensive overview of recent progress in polymer and nanofiber materials for energy storage, with an emphasis on sustainable design, functional enhancement, and future directions enabled by bioengineering approaches, an overview of which is presented in Figure 1.

## 2. Nanofibers in Lithium-Ion Batteries (Li-Ion)

Electrospinning has emerged as a robust and scalable technique for fabricating nanofibers from a wide range of polymer solutions and melts through the application of a high-voltage electric field. This method is distinguished by its simplicity and versatility, enabling the production of continuous fibers with diameters spanning from tens of nanometers to a few micrometers. In previous studies [1,2], we and others have demonstrated that electrospinning enables precise control over nanofiber morphology and performance characteristics, making it highly suitable for functional material development.

The electrospinning process involves dispensing a polymer solution through a syringe fitted with a metallic needle, across which a high voltage is applied relative to a grounded collector. As the electric field strength increases, electrostatic forces exceed the surface tension at the droplet’s apex, initiating the formation of a charged polymer jet. This jet undergoes elongation and complex whipping instabilities during its flight, resulting in the deposition of ultrafine fibers onto the collector surface.

**Figure 2:**
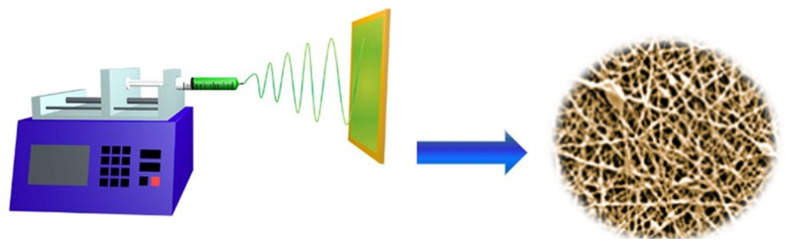
Electrospinning process, graphical presentation of transforming liquid polymer into nanofibers of desired properties.

The resultant nanofiber mats exhibit advantageous structural features, including high surface-area-to-volume ratios, tunable porosity, and customizable morphology. These attributes render electrospun fibers particularly attractive for advanced applications in energy storage, catalysis, environmental remediation, biomedical engineering, and filtration technologies. Moreover, the electrospinning process offers extensive parameter space for optimization: polymer concentration, solution viscosity, applied voltage, flow rate, needle-to-collector distance, and ambient conditions (e.g., temperature, humidity) can all be modulated to tailor fiber properties for specific end-use applications (Figure 2) [1].

In our research, we have extensively investigated the electrospinning process as a reliable and tunable method for fabricating nanofibrous materials tailored for energy storage applications. Our findings demonstrate that by carefully adjusting processing parameters—such as voltage, flow rate, polymer concentration, and ambient humidity—it is possible to finely control fiber diameter, morphology, and porosity. Specifically, we observed that lower polymer concentrations tend to produce bead-on-string structures, while optimized concentrations yield uniform, bead-free nanofibers with diameters ranging from 100 to 400 nm. We also found that incorporating functional nanoparticles or bio-derived additives into the spinning solution enhances the electrochemical properties of the resulting nanofibers without compromising their structural integrity.

To visually summarize the transformation of a liquid polymer solution into solid nanofibers, we have developed a schematic illustration (Figure 2) of the electrospinning setup. The graphic shows a syringe loaded with polymer solution connected to a high-voltage power supply, forming a Taylor cone at the needle tip. The charged polymer jet is then ejected, undergoes elongation and whipping instabilities, and deposits as a nonwoven mat of nanofibers on the grounded collector. This process enables the continuous production of fibers with large surface-area-to-volume ratios and tunable chemical functionality, making them ideal candidates for separators and electrodes in lithium-ion batteries and other electrochemical systems, just to mention some of the possible applications.

Polymers offer a suite of advantages that make them highly attractive for modern energy storage technologies. One of the most notable features is their intrinsic mechanical flexibility, which supports the fabrication of flexible, wearable, and stretchable energy storage devices—an essential requirement for emerging applications in next-generation electronics and portable systems. In particular, electrospun polymer nanofibers demonstrate exceptional chemical tunability, enabling surface functionalization strategies that can improve ionic conductivity, mechanical robustness, and electrochemical stability.

The low density of polymeric materials further contributes to enhanced energy system performance by minimizing overall device weight, a critical consideration in mobile and aerospace technologies. Importantly, the solution processability of many polymers facilitates scalable and cost-efficient manufacturing, aligning with industrial demands for high-throughput production.

**Figure 3:**
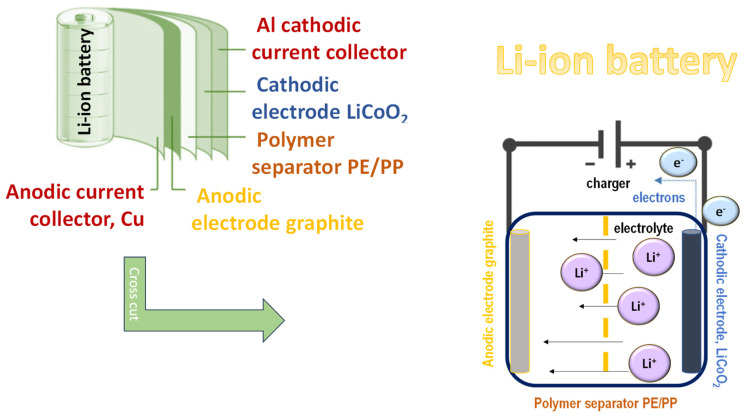
Schematic overview of Li-ion battery with polymer separator made by electrospinning.

Electrospun nanofibers have garnered widespread attention for use as separators and electrodes in lithium-ion batteries, where their high surface area, interconnected porous structure, and tunable morphology provide enhanced ion transport and electrode–electrolyte interactions (Figure 3). These structural and functional advantages position polymer-based nanofibers as pivotal materials in the development of high-performance and sustainable energy storage devices.

Advancements in bioengineering have enabled the development of genetically modified protein-based fibers, such as recombinant silk fibroin and bacterial cellulose, for use in battery separators. These bioengineered nanofibers offer several key benefits, including enhanced thermal stability, increased ionic conductivity, and inherent self-healing properties. By modifying amino acid sequences or incorporating specific functional motifs, such fibers can be tailored to present chemical groups that selectively interact with lithium ions, thereby promoting efficient ion transport and reducing interfacial resistance.

This molecular-level customization has been shown to improve charge/discharge rates, extend cycle life, and enhance the overall safety of lithium-ion batteries. As a result, genetically engineered protein-based nanofibers are emerging as promising materials for next-generation bio-integrated energy storage systems, combining performance enhancement with improved structural and functional adaptability [1,2,3].

## 3. Nanofibers in Supercapacitor Electrodes

Polymers have emerged as essential materials in the advancement of next-generation energy storage technologies, particularly due to their ability to enhance ionic conductivity, thermal stability, and cycling performance. These functional properties are central to enabling scalable, low-cost, and environmentally sustainable alternatives to traditional inorganic materials. Beyond their performance benefits, polymeric materials—especially those derived from renewable or biodegradable sources—are increasingly viewed as pivotal enablers of a circular economy in energy systems. The design of such materials supports closed-loop life cycles, reduced environmental impact, and greater alignment with green manufacturing initiatives.

While synthetic polymers, including polyethylene oxide (PEO), polyvinylidene fluoride (PVDF), and polyaniline (PANI), currently dominate commercial applications due to their established performance and processability, increasing societal and regulatory pressures have catalyzed significant interest in bio-derived alternatives. Materials such as chitosan, nanocellulose, lignin, and silk fibroin are now under extensive investigation for their potential to serve as electrolytes, separators, and electrode matrices. Recent progress in functionalization techniques and nanostructuring approaches—particularly through blending, doping, or forming hybrid composites—has further expanded the performance envelope of these biopolymers.

**Figure 4:**
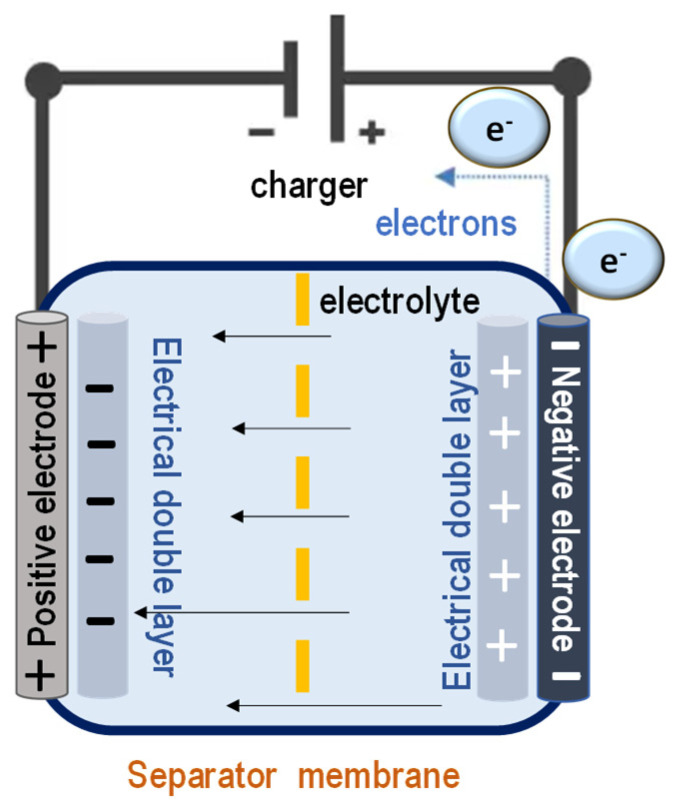
Supercapacitor electrode.

Supercapacitors, as a key class of energy storage devices, exemplify the potential of polymer integration. They offer a unique balance between the high energy density of batteries and the rapid charge–discharge characteristics of traditional capacitors. A standard supercapacitor configuration includes two electrodes, a porous ion-conducting separator, and an electrolyte medium (Figure 4). Polymers play a critical role in each of these components, whether as binders, conductive frameworks, or separator membranes. Their high surface area, chemical tunability, and flexibility are especially advantageous for the development of wearable, flexible, and miniaturized devices. As interdisciplinary efforts in materials science, synthetic biology, and process engineering converge, polymer-based supercapacitors are positioned to drive sustainable and efficient energy storage technologies into mainstream applications.

Supercapacitors are effective alternatives to traditional batteries and capacitors, combining features of both. The separator must resist electrical current while enabling high ion conductivity and permeability. Specific configuration of supercapacitor cells enables them to bridge the gap between electrochemical batteries, known for their energy storage capabilities, and conventional capacitors, which excel in rapid energy discharge. The porous separator plays a critical role in this setup—it must exhibit high electrical resistance to prevent current leakage while simultaneously offering high ionic conductivity to facilitate the smooth transport of charged ions. Additionally, it must be permeable to ions to enable efficient charge movement between electrodes.

What set supercapacitors apart was their unique ability to combine high power density with relatively high energy density. Power density referred to how quickly a system could deliver energy relative to its mass or volume, which was essential for applications demanding rapid bursts of energy. On the other hand, energy density measured the total amount of energy the system could store, making it important for long-duration applications. In simple terms, power density represented the system’s responsiveness, while energy density indicated its endurance in storing energy.

Beyond just these two metrics, supercapacitors possess a range of unique properties that enhance their appeal across diverse applications. They are known for their long operational lifespans, wide temperature tolerance, affordability, and minimal environmental impact. These qualities have contributed to their growing usage in numerous sectors. For example, they are employed in energy-harvesting systems such as wind turbines, contribute to power grid stabilization, and are integral to uninterruptible power supply (UPS) systems. In the automotive industry, supercapacitors play a vital role in applications like regenerative braking systems and emergency door operations. Furthermore, their reliability and rapid response make them ideal for use in electronic devices as backup memory storage solutions.

Conductive polymers, including polyaniline (PANI), polypyrrole (PPy), and poly(3,4-ethylenedioxythiophene) (PEDOT), were widely utilized in electrode materials due to their excellent electrical conductivity and inherent redox activity. In lithium-ion batteries (LIBs), polymeric electrolytes such as polyethylene oxide (PEO) and polyvinylidene fluoride (PVDF) played a key role in facilitating ionic transport while retaining mechanical flexibility. Furthermore, polymeric separators like polypropylene (PP) and polyethylene (PE) were essential for maintaining battery safety by effectively preventing internal short circuits.

In contrast, biopolymers such as nanocellulose and chitosan have attracted significant interest as eco-friendly alternatives for use as electrolytes and separators, owing to their biodegradability, film-forming capability, and inherent ionic conductivity. Other biopolymers, including lignin and polypeptides, have been investigated for use in redox-active electrodes, offering renewable substitutes to conventional synthetic conductive polymers. Despite their environmental advantages, these materials still face notable limitations, including low intrinsic electrical conductivity, sensitivity to moisture, and limited electrochemical stability. To overcome these drawbacks, researchers have focused on enhancing their performance through structural modifications and the development of composites with carbon nanomaterials or metal nanoparticles.

While polymer-based energy storage materials have shown great promise, they encounter several critical challenges. One such challenge is their low intrinsic conductivity, as even conductive polymers often require doping or hybridization with inorganic materials like graphene or carbon nanotubes to enhance their conductivity. Additionally, thermal and chemical stability remain significant concerns, as many polymers degrade at high temperatures or in harsh electrochemical environments. Polymer electrolytes also tend to suffer from lower ionic conductivity compared to liquid electrolytes. Environmental concerns are another issue, as synthetic polymers contribute to waste accumulation, while biopolymer-based alternatives face scalability challenges.

To address these limitations, recent research has focused on several innovative approaches. One area of development is the creation of hybrid polymer composites, which combine polymers with ceramics, ionic liquids, or nanostructured carbons to improve stability and conductivity. Another advancement is the use of polymer electrolytes in solid-state batteries, where next-generation solid-state lithium and sodium batteries aim to replace liquid electrolytes with safer polymer-based alternatives. Research into self-healing and shape-memory polymers is also underway to enhance the lifespan and durability of energy storage materials. Furthermore, the exploration of biodegradable polymer electrodes, particularly biopolymer-derived carbon materials, holds promise for creating sustainable battery electrodes.

Combining batteries and supercapacitors into hybrid devices requires materials with both high energy and power densities. Genetically modified nanofibers are being used to bridge this gap by serving as multifunctional scaffolds with embedded electroactive moieties. These biofibers offer the dual benefit of structural support and electrochemical activity, enabling improved charge retention and cycling stability in hybrid systems.

Table 1 shows the comparison of synthetic and biopolymer-based materials from the perspective of energy storage applications.

Biopolymers made by genetic modification, a biotechnological process in which specific genes are isolated, manipulated, and transferred into another living organism to alter or enhance particular traits, are very interesting materials. Such targeted modification of the organism’s genetic makeup enables the creation of new biological materials with improved or entirely novel properties, useful in the engineering of novel strains of microorganisms that are able to produce specific enzymes used in polymer processing. In the textile industry, genetically modified (GM) organisms have been widely utilized to develop cotton crops that are pest-resistant without the need for pesticides, produce naturally colored cotton without synthetic dyes, and even generate silk with enhanced attributes—such as fluorescence or altered morphology—derived from transgenic modifications involving organisms like potatoes, tomatoes, goats, and bacteria.

Notably, genes responsible for desirable traits such as tensile strength, elasticity, and thermal stability have been transferred from species like spiders into other hosts, such as sheep or goats, to produce high-performance proteins that can be harvested and processed into advanced fibers. These breakthroughs pave the way for creating novel fiber materials that combine the benefits of biotechnology with traditional and modern manufacturing techniques, including electrospinning.

The scientific foundation of genetic engineering is rooted in the understanding of genes as the basic units of heredity, a concept first demonstrated by Gregor Mendel in the 19th century through his work with garden pea plants. Mendel’s experiments revealed that traits are inherited in predictable patterns, laying the groundwork for the modern understanding of genes as segments of DNA located on chromosomes. Building on this knowledge, advances in molecular biology have enabled the precise manipulation of genetic material.

Incorporating genetic modification into the design of nanofibers opens new avenues for innovation in energy storage. By embedding genetically engineered proteins into electrospun nanofibers, researchers can create materials with tailored properties such as enhanced conductivity, mechanical strength, and chemical functionality. These genetically modified (GM) nanofibers offer significant potential for next-generation lithium-ion batteries, supercapacitors, and hybrid systems—where performance, longevity, and sustainability are critical requirements.

Since the elucidation of DNA’s double-helix structure by Watson and Crick in 1953, molecular genetics has laid the groundwork for transformative advances in genetic engineering. By manipulating nucleotide sequences, scientists have developed methods to edit genomes across diverse organisms, enabling the creation of genetically modified organisms (GMOs) with tailored biological functions. Milestones such as Paul Berg’s development of recombinant DNA technology and the discovery of *Agrobacterium tumefaciens* as a natural gene-transfer agent in plants have propelled gene editing into mainstream applications, particularly in agriculture and material science.

Early GMO applications focused on traits such as pest resistance and abiotic stress tolerance. Today, advances in genomics and synthetic biology enable more sophisticated modifications—ranging from allergen-free crops to plants expressing industrially relevant compounds. In the context of sustainable materials, this progress has extended into textiles and functional fibers.

Genetically engineered protein-based materials—such as recombinant silk fibroin, elastin-like polypeptides (ELPs), and amyloid fibrils—are now being leveraged in the fabrication of nanofibers with customizable mechanical, electrical, and electrochemical properties. When processed via electrospinning, these engineered biomolecules can form nanostructures that enhance ionic conductivity, thermal stability, and mechanical strength—key attributes for high-performance components in lithium-ion batteries and supercapacitors.

Crucially, these bioengineered nanofibers offer molecular programmability, allowing surface functionality and redox behavior to be tailored for specific energy applications. Hybridization with conductive polymers or carbon-based materials further boosts charge storage capacity and cycling stability, positioning GM-derived nanofibers as a sustainable and scalable alternative to conventional energy storage materials.

### 3.1. Genetically Modified Cotton (GM Cotton)

One of the most extensively explored genetically modified (GM) fibers is cotton, primarily due to its global importance and susceptibility to pest damage. A prominent example of genetic engineering in cotton is the development of insect-resistant varieties through the insertion of a gene from *Bacillus thuringiensis* (Bt), which enables the plant to synthesize an insecticidal protein. This Bt cotton has demonstrated a reduction in pesticide usage by up to 75%, offering a dual advantage: effective pest control and the preservation of beneficial insect biodiversity within the ecosystem [4].

In the United States, GM cotton now accounts for approximately 71% of total cotton cultivation, a testament to its economic and agronomic viability [5]. However, challenges persist. Some field trials have reported that Bt cotton is ineffective against secondary pests, which were unaffected by the introduced gene, raising concerns about its long-term ecological impact and sustainability [6].

Another promising direction in GM cotton research is the development of naturally pigmented cotton fibers, which aim to eliminate the need for chemical dyes. Through nuclear magnetic resonance (NMR) analysis, researchers have identified various flavonoid anthocyanins (types 1–5) responsible for color variations in cotton fibers, ranging from red and purple to green and blue. This innovation could streamline post-harvest processing, reduce water and chemical use, and produce more sustainable textiles [7].

Nevertheless, there are biosafety concerns associated with genetically engineered cotton. For instance, certain varieties such as Bollgard and Roundup Ready cotton have exhibited resistance to antibiotics like streptomycin and spectinomycin. The potential transfer of these resistance traits—through contact with medical dressings or hygiene products—raises the risk of promoting antibiotic-resistant pathogens in human populations, such as *Neisseria gonorrhoeae*.

### 3.2. Genetically Modified Wool (GM Wool)

In the domain of animal fibers, genetically modified wool represents a promising avenue for producing textiles with enhanced properties. Australian researcher Tony Schlink and his team have demonstrated that traits such as wool shrinkage are heritable and can be targeted through selective breeding or genetic modification. Wool produced from genetically optimized sheep yields longer, stronger yarns with improved resistance to felting and tangling during the spinning process [8].

Genetic studies have also indicated that dust-retention—a significant concern in textile hygiene and performance—is partially governed by the sheep’s genome. This opens the door to the development of sheep breeds whose wool resists dust accumulation, offering functional benefits in both consumer and industrial textile applications.

### 3.3. Genetically Modified Silk (GM Silk)

Silk, known as the strongest natural fiber, has long fascinated researchers due to its unique mechanical properties. In particular, spider silk and silkworm silk exhibit high tensile strength and elasticity, making them ideal candidates for biomimetic fiber engineering. American researchers have been actively studying the protein regulation mechanisms within the silk glands of spiders and silkworms. These glands finely control protein concentration and water content, thereby influencing the physical properties of the silk fibers—variations that differ significantly across spider species [8,9,10,11,12,13,14,15,16,17,18,19,20].

Despite the difficulty in studying spider silk at the nanoscale (diameter ~2.5 × 10⁻^8^ m), advances in bioengineering have enabled the recombinant production of silk proteins in genetically modified organisms such as bacteria, yeast, and even goats. These silk-based nanofibers hold tremendous promise for energy applications due to their molecular regularity, high surface area, and mechanical robustness, making them suitable candidates for reinforcing polymer composites, bio-batteries, and smart textiles.

### 3.4. Spider Silk Biopolymer (“Bio-Steel”)

Spider silk is composed of protein filaments—fibroin and sericin—which are themselves constructed from long chains of amino acids, with glycine and alanine being the most abundant. The resulting fiber, known as spider silk, exhibits remarkable mechanical properties: it is five times stronger than steel of the same diameter and twice as strong as Kevlar. In addition to strength, spider silk is highly elastic, capable of stretching two to four times its original length without breaking. Furthermore, it is both water-resistant and retains its structural integrity even at temperatures as low as –40 °C [21].

Through genetic recombination, scientists have successfully combined genes from the orb-weaving spider (*Araneus diadematus*) with the Nigerian dwarf goat, enabling the production of silk proteins in the goat’s milk. Once fats, milk proteins, and water are removed, a silken slurry remains—composed of fine, fibrous threads [22,23,24].

Some genetic modifications arise unintentionally. For instance, Japanese scientists, during protein expression trials in insects, accidentally produced fluorescent silk threads that glow in the dark—a result of random gene insertion leading to the expression of fluorescent proteins in silkworm silk [25]. Today, numerous patents exist on spider silk-related genes and recombinant technologies, several of which are summarized in Table 2.

Recombinant biopolymers such as genetically engineered spider silk—often termed “bio-steel”—exhibit a unique combination of high tensile strength, elasticity, biocompatibility, and lightweight architecture. These properties make them highly attractive across a wide range of applications, including medical sutures, bone scaffolds, military-grade armor, aerospace components, and high-resilience automotive materials. Efforts to scale production have moved beyond transgenic animals (e.g., goats) toward bacterial, plant, and other mammalian expression systems, offering more sustainable and controllable platforms.

In the context of energy storage, genetically modified protein-based fibers are gaining traction as multifunctional components in composite electrodes and flexible electronics. Engineered silk fibroins, keratins, and celluloses, when functionalized with conductive domains or blended with graphene and metal oxides, enable the design of electrodes that combine mechanical integrity with enhanced ionic conductivity and thermal resilience. These materials offer distinct advantages in developing biocompatible, biodegradable systems—especially for wearable and implantable power sources.

Moreover, such fibers are increasingly applied in triboelectric nanogenerators (TENGs), where surface charge tunability and mechanical compliance are critical. Recombinant keratin and chitosan nanofibers, for instance, have demonstrated enhanced triboelectric output, positioning them as promising candidates for self-powered biosensors and energy-harvesting devices.

Despite their promise, genetically engineered fibers raise environmental and ethical concerns, particularly regarding long-term ecological impacts and biosafety. While applications in high-value sectors such as medicine and defense are more established, their broader adoption in energy storage systems remains constrained by production scalability, stability under electrochemical conditions, and regulatory uncertainties. Nonetheless, continued interdisciplinary efforts may unlock their potential as sustainable alternatives in next-generation energy technologies.

## 4. Nanofibers in Batteries and Fuel Cells

Nanofibers produced from genetically modified sources, such as recombinant silk or lignin derivatives, have been developed as coatings for separators and solid-state electrolytes. These materials enhance thermal tolerance and suppress dendritic growth while maintaining high ion conductivity. Their bio-origin allows for renewable sourcing and potential biodegradability, contributing to the eco-friendliness of the overall device.

### 4.1. Nanofibers in Sodium-Ion Batteries

Sodium-ion batteries (SIBs) benefit from the open structure and functional adaptability of bioengineered nanofibers. Genetically modified bacterial cellulose, for instance, offers a highly porous and mechanically resilient matrix for sodium storage. Modifying these fibers at the genetic level to express functional groups tailored to Na⁺ ions can improve interfacial compatibility and storage efficiency, making SIBs more viable as sustainable alternatives to Li-ion systems.

### 4.2. Nanofibers in Fuel Cells

**Figure 5:**
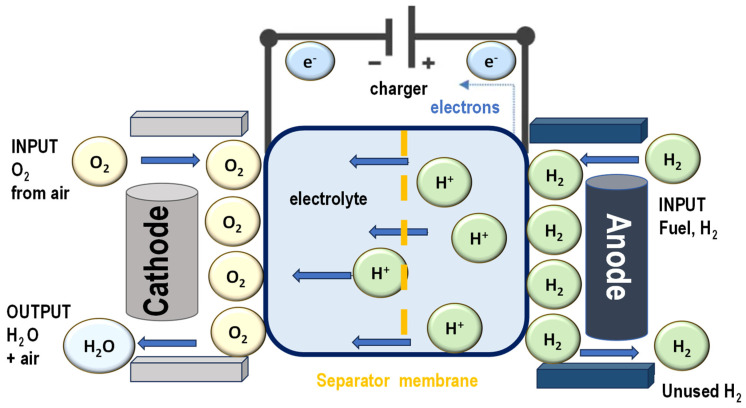
PEM fuel cell schematic overview where the PEM fuel cell system operates under transient conditions, assuming the gas mixture behaves as a perfect gas and that the incoming gas mixture is treated as an incompressible fluid.

Proton exchange membrane (PEM) fuel cells are advanced electrochemical energy conversion devices that generate electricity through redox (reduction–oxidation) reactions involving hydrogen and oxygen. These fuel cells work by splitting hydrogen molecules at the anode, where each molecule is separated into protons and electrons. The protons pass through the proton exchange membrane, while the electrons travel through an external circuit, producing an electric current that can power various devices or systems. At the cathode, the protons, electrons, and oxygen combine to form water, which is the only by-product of the reaction (Figure 5).

PEM fuel cells are particularly valued for their high energy conversion efficiency, which allows for more effective use of the chemical energy stored in hydrogen compared to combustion-based systems. They are also highly reliable, offering consistent performance over extended periods. Importantly, since their only emissions are heat and water, PEM fuel cells produce no pollution at the point of operation, making them an environmentally friendly alternative to fossil fuel-based energy sources.

These fuel cells are used across a wide range of applications due to their clean operation, compact size, and quick start-up capabilities. In the transportation sector, they are increasingly utilized in fuel cell electric vehicles (FCEVs), including cars, buses, and even trains. In stationary applications, they provide backup and off-grid power solutions for buildings, data centers, and other infrastructure. Additionally, they are gaining popularity in portable power systems for military and emergency services, as well as for personal electronic devices.

The design of PEM fuel cells is mechanically simple, featuring no moving parts in the electrochemical stack. This structural simplicity contributes to their enhanced durability, reduced maintenance requirements, and longer operational lifespan. These advantages, combined with ongoing advancements in materials and manufacturing processes, continue to drive the development and deployment of PEM fuel cells as a key component of a cleaner and more sustainable energy future.

As mentioned before, electrospinning is a promising technique for combining biopolymers with synthetic polymers and for obtaining nanofibers effective in energy storage and conversion [26,27,28,29,30].

Shi et al. proved that electrospinning is an efficient and cost-effective method for producing nanofibers (NFs) with unique properties beneficial for fuel cell applications. In particular, electrospun NFs used in direct methanol fuel cells (DMFCs) demonstrated enhanced performance due to their high surface-to-volume ratio and tunable morphology. However, challenges such as methanol crossover through the proton exchange membrane (PEM) remain critical issues [30]. In addition, the authors have emphasized that future research should focus on developing durable and redox-active cathode catalysts and improving PEM materials to achieve efficient and stable fuel cell systems. This is in correlation to Zhu et al. [31], who confirmed that electrospun nanofibrous materials are excellent in addressing numerous issues, especially in energy fields.

The high surface area, porosity, and tunable morphology of electrospun nanofibers enhance ion conductivity and facilitate effective gas and water transport, which are critical for optimal fuel cell performance. These fibers can be engineered to incorporate functional materials, such as phosphonated polymers, sulfonated compounds, or metal nanoparticles, improving proton exchange membrane efficiency and mechanical durability [31,32,33]. Recent studies have demonstrated significant advancements using electrospun membranes in proton exchange membrane fuel cells (PEMFCs), offering enhanced performance and longevity compared to conventional membranes. As research progresses, electrospinning continues to enable innovative fuel cell designs by providing flexible, scalable, and customizable nanostructures that meet the evolving demands of clean energy technologies [34,35,36]. López-Covarrubias proposed application in solar sensitive cells [35], while Nadaf et al. listed several possible applications, such are fuel cells, disc electrodes, dye-sensitized solar batteries or cells, solar batteries, and energy retention devices [36].

Moreover, electrospinning plays a pivotal role in advancing both lithium-ion and sodium-ion battery technologies by enabling the fabrication of nanostructured materials with superior electrochemical properties. For lithium-ion batteries (LIBs), electrospun nanofibers are employed to create high-performance anodes, cathodes, and separators, offering benefits such as enhanced conductivity, large surface area for ion diffusion, and robust mechanical integrity. Materials like electrospun carbon, silicon composites, and polymeric nanofibers have shown remarkable improvements in cycle stability, capacity retention, and rate performance [37,38]. More precisely, Ren et al. investigated nanofibers as free-standing cathodes for ultralong-life and high-rate sodium-ion batteries, and reported that they have successfully synthesized NFPP/C nanofibers using a straightforward electrospinning followed by a carbonization process. When tested as a cathode material, the nanofibers exhibited outstanding rate capabilities, delivering specific capacities of 118 mA h g^−1^ at 0.2 C and 64 mA h g^−1^ at 20 C. Remarkable cycling stability was also demonstrated, with 79.6% capacity retention over 10,000 cycles at 10 C and no significant voltage decay. To evaluate practical applicability, a full cell was assembled and tested, achieving a reversible capacity of 126.4 mA h g^−1^ [37]. Such properties could be enhanced by flexible fuel cells made by Wang [38].

Similarly, in sodium-ion batteries (SIBs), which are gaining traction due to the abundance and low cost of sodium, electrospun materials are used to overcome challenges related to the larger ionic radius and slower diffusion kinetics of sodium ions. Free-standing electrospun cathodes and structured anode materials improve the structural stability and energy density of SIBs, making them viable alternatives for large-scale energy storage. Overall, electrospinning offers a scalable and customizable approach to designing next-generation batteries with improved safety, performance, and longevity. Huang et al. prepared a review on tailoring the structure of silicon-based materials for lithium-ion batteries via electrospinning technology [39] with defining limitations of such solutions were clearified as silicon anodes have their shortcomings, such as low electrical conductivity and tremendous volume changes during lithium-ion intercalation/deintercalation, posing a series of challenges to their commercialization. However, Chen et al. provided solution for facile design for stable lithium metal anodes to promote the practical use of LMBs and other alkali metal batteries by lacing an electroactive polyvinylidene fluoride/polymethyl methacrylate (PVDF/PMMA) composite nanofiber interlayer on a current collector, inducing uniform lithium deposition to mitigate the dendrite problem [40].

Carbon nanofibers (CNFs), typically produced via electrospinning of polymer precursors such as polyacrylonitrile (PAN), polyimide, lignin, or pitch, followed by thermal stabilization and carbonization, have become indispensable in next-generation energy storage and conversion technologies. The electrospinning process affords fine control over fiber morphology, diameter, porosity, and alignment, enabling tailored properties for diverse applications [41,42,43].

In lithium-ion and sodium-ion batteries, electrospun CNFs serve as efficient anode and cathode materials or conductive scaffolds. Their continuous fibrous networks support rapid electron transport and provide extensive active sites for ion intercalation. The high porosity and interconnected structure facilitate superior electrolyte infiltration and ionic diffusion, improving rate capabilities and cycling stability. Additionally, CNFs have been employed as binder-free current collectors, enhancing mechanical strength and conductivity without metal substrates [44].

Beyond conventional batteries, CNFs play a critical role in lithium–sulfur (Li–S) and lithium–air (Li–O_2_) batteries. In Li–S systems, they act as sulfur hosts that immobilize polysulfide intermediates, mitigating the shuttle effect and thus enhancing coulombic efficiency and cycle life. For Li–O_2_ batteries, electrospun CNFs provide porous, conductive air electrodes that catalyze oxygen redox reactions, supporting high energy density and rechargeability. Hybrid composites combining CNFs with metal oxides or sulfides further improve electrochemical performance through synergistic effects [45,46,47].

Supercapacitors also benefit from electrospun CNFs, which offer rapid charge/discharge rates, high power density, and extended durability. Heteroatom doping (e.g., N, S, P) enhances pseudocapacitive behavior and energy density, while the intrinsic flexibility and low weight of CNF mats suit wearable and portable electronics [46].

In addition to storage devices, CNFs serve as conductive frameworks and catalyst supports in electrocatalysis and fuel cells. Their tunable morphology and scalable fabrication enable multifunctional energy materials that are both cost-effective and high-performing [48,49,50]. For example, genetically engineered nanofibers functionalized with proton-conducting groups (sulfonic acid, imidazole) show promise as ion-conductive channels or catalyst supports in proton exchange membrane (PEM) fuel cells, potentially reducing costs and enabling greener synthesis routes.

### 4.3. Green Sustainable and Environmental Applications

Electrospun nanofibers have emerged as versatile materials addressing key environmental challenges, particularly in carbon dioxide (CO_2_) reduction and air pollutant remediation. Their high surface-area-to-volume ratio, tunable porosity, and structural flexibility render them ideal platforms for catalytic and adsorptive applications [51]. In CO_2_ reduction, electrospun mats provide efficient supports for photocatalysts and electrocatalysts such as metal oxides (e.g., TiO_2_, ZnO), metal–organic frameworks (MOFs), and doped carbon materials. For example, reduced graphene oxide (rGO)-wrapped Ag-doped TiO_2_ nanofibers demonstrate enhanced visible-light photocatalytic activity, converting CO_2_ into hydrocarbons like methane and methanol by facilitating improved light absorption, charge separation, and reactant–catalyst interaction [52].

Beyond carbon capture, electrospun fibers serve critical roles in air purification. Their intricate networks physically trap particulate matter (PM2.5, PM10) while enabling chemical adsorption of volatile organic compounds (VOCs) and gases such as NOₓ and SO_2_. Functionalization with activated carbon, photocatalytic nanoparticles, or antimicrobial agents further enhances pollutant removal efficiency, enabling integration into wearable filtration devices and energy-efficient HVAC systems [53].

In water treatment, electrospun nanofibers remove heavy metals, dyes, and pharmaceuticals via adsorption and catalytic degradation. They are also increasingly incorporated into environmental sensors, with embedded responsive materials allowing real-time pollutant detection. The possibility of eco-friendly fabrication using biodegradable or recycled polymers further strengthens their sustainability credentials.

Overall, electrospinning stands out as a powerful and adaptable fabrication technique for producing advanced nanofiber materials that contribute meaningfully to environmental protection, pollution control, and carbon neutrality initiatives.

### 4.4. Electrospun Materials as Anodes, Separators, and Electrolytes

As anodes, electrospun nanofibers, particularly those made from carbon, tin, or silicon, provide excellent conductivity and structural stability, which is crucial for enhancing the charge/discharge cycles and capacity of Li-ion batteries. Electrospun separators, made from materials like polyacrylonitrile (PAN) or polyvinylidene fluoride (PVDF), act as critical components to prevent short circuits while allowing efficient ion transport between the anode and cathode, thus ensuring safe and stable battery operation. Electrospun polymer electrolytes, such as PVDF-based fibrous membranes, further improve the ionic conductivity and mechanical integrity of the battery, especially in solid-state systems where liquid electrolytes are replaced by solid polymer materials. These electrospun materials provide a more robust, flexible, and lightweight alternative to traditional battery components, improving the overall performance and longevity of energy storage devices [54,55,56,57,58,59,60].

The development and transformation of electrospun carbon nanofibers (CNFs) involve a series of processes that can significantly influence their performance, especially for energy storage, catalysis, and environmental applications. Fabrication begins with electrospinning, where polymeric precursors like polyacrylonitrile (PAN), lignin, or polyimide are dissolved in a solvent and subjected to an electric field. This process forms nanofibers that possess high surface area and porosity, which are crucial for applications like supercapacitors, lithium-ion batteries, and fuel cells. The resulting polymeric nanofibers are then subjected to carbonization, typically under inert conditions such as nitrogen or argon, at high temperatures to convert the organic fibers into carbon structures. This transformation improves their electrical conductivity and thermal stability, making them suitable for energy storage devices. Polyacrylonitrile was widely investigated as a promising polymer for nonwoven separators in Li batteries and other fuel cells [59,60,61,62,63]. Zhou developed carbon nanofibers by using aligned electrospun polyacrylonitrile (PAN) nanofiber bundles [59]. Cho focused on investigating the thermal stability of PAN-based nanofibers, specifically evaluating their potential use as nonwoven separators in lithium-ion batteries [60]. Building on these findings, Rahaman provided a comprehensive review of how heat treatment affects the structure and performance of PAN fibers, highlighting its significance in enhancing their properties for energy storage applications [61].

To further enhance the properties of electrospun carbon nanofibers, post-treatment processes, such as activation, doping, and functionalization, are often employed. Activation typically involves exposing the carbon nanofibers to oxidative agents or high-temperature treatments to introduce porosity, which increases the surface area and enhances their performance in energy applications. Doping with heteroatoms, like nitrogen, boron, or sulfur, can further improve their electrochemical properties, making them highly effective in energy storage systems like lithium-ion or sodium-ion batteries. Moreover, functionalization of CNFs can introduce specific surface groups that enhance their interaction with other materials, such as catalysts or electrodes in fuel cells and batteries. Through these methods, electrospun carbon nanofibers can be tailored to meet the specific requirements of various advanced technological applications, offering great potential for the development of high-performance, durable, and efficient materials in the field of energy storage and conversion [64,65].

### 4.5. Advances in Super Capacitors and Fuel Cells

Supercapacitors, known for their ability to deliver rapid bursts of energy with long cycle life, rely heavily on materials with high surface area, good conductivity, and excellent mechanical stability. CNFs, derived through electrospinning, are highly suited for these applications due to their excellent structural characteristics, such as high surface area, porosity, and conductivity [66,67,68,69,70].

The electrospinning process enabled the fabrication of nanofiber mats with controlled morphologies, which were critical for enhancing the electrochemical performance of supercapacitor electrodes. The resulting nanofiber networks offered a highly accessible surface area for ion storage and supported efficient ion transport—both key factors in achieving rapid charge and discharge rates. Additionally, the large surface area of the electrospun carbon nanofibers contributed to a higher charge storage capacity per cycle, thereby improving the overall energy and power density of the supercapacitors [59,63,65]. Ruiz Rosas et al. have successfully produced submicron-diameter carbon fibers by the electrospinning of lignin [67]. To further enhance the performance of electrospun CNF-based electrodes, additional modifications can be applied. For example, the carbon nanofibers can be activated or doped with heteroatoms such as nitrogen, oxygen, or sulfur to increase their electrochemical properties, like specific capacitance and conductivity. Furthermore, hybrid structures where carbon nanofibers are combined with other materials, such as conductive polymers, graphene, or metal oxides, can significantly improve charge storage and cycling stability. These hybrid composites can take advantage of the synergistic properties of each component, leading to electrodes that perform well across a wider range of applications. The flexibility and scalability of electrospinning, combined with the tunable properties of carbon nanofibers, make them an ideal candidate for the next generation of energy storage devices, particularly supercapacitors that require both high power density and long operational lifetimes [71,72,73].

### 4.6. Graphene, CNTs, and Nanocomposites for Batteries

Graphene, carbon nanotubes (CNTs), and nanocomposites have garnered significant attention as advanced materials for use in batteries, particularly lithium-ion and sodium-ion batteries, due to their exceptional electrical conductivity, mechanical strength, and large surface areas [74,75,76,77,78,79,80,81,82,83,84]. These materials play a critical role in enhancing the performance of battery electrodes by improving charge storage capacity, cycling stability, and energy efficiency. Yoo et al. demonstrated that lithium storage in layered graphene-based compounds is significantly affected by the interlayer spacing between graphene nanosheets. Their study highlighted that adjusting this spacing using interacting molecules such as carbon nanotubes (CNTs) or fullerenes (C60) can play a critical role in enhancing the overall storage capacity. Specifically, they reported a specific capacity of 540 mAh/g for pristine graphene nanosheets (GNSs), which notably exceeds that of conventional graphite. Furthermore, by incorporating CNTs and C60 macromolecules into the GNS structure, the specific capacity was further increased to 730 mAh/g and 784 mAh/g, respectively, showcasing the potential of molecular engineering in improving lithium-ion battery performance [77].

Graphene is a single layer of carbon atoms arranged in a hexagonal lattice, known for its remarkable electrical, thermal, and mechanical properties. Its large surface area and high conductivity make it an excellent candidate for anode and cathode materials in batteries.

Graphene aerogels (GAs) are among the lightest solid materials known, garnering significant attention in both academic research and industrial applications due to their extraordinary combination of properties. These synthetic materials are characterized by their ultra-low density, high porosity, and a unique three-dimensional network structure that endows them with remarkable mechanical strength, excellent electrical conductivity, superior thermal stability, and impressive adsorption capabilities.

**Figure 6:**
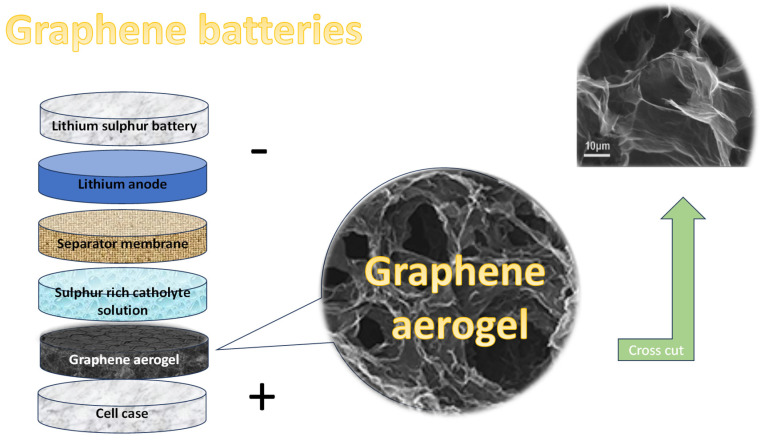
Graphene-based battery, schematic overview.

The fabrication of graphene aerogels generally starts with a graphene oxide (GO) solution, which is reduced—either chemically or thermally—to form a hydrogel composed of interconnected graphene sheets. To maintain the porous structure of this hydrogel, the water content is typically removed through freeze-drying, a method that avoids structural collapse by sublimating ice directly into vapor. The result is a lightweight, dry aerogel with a highly porous architecture, such is presented in Figure 6.

Graphene aerogels are attractive for a wide range of advanced applications due to their unique combination of low density, high surface area, electrical conductivity, and mechanical strength. They have demonstrated significant promise in energy storage devices like batteries and supercapacitors, as well as in environmental cleanup, thermal insulation, sensing technologies, and catalysis. Moreover, their structure can be tuned and easily combined with other functional materials, making them highly adaptable for next-generation technologies.

Comparison of the application of the different abovementioned nanofibers in energy storage is presented in Table 3.

Graphene-based materials enable efficient electron transport and ion diffusion within the electrode, which significantly enhances the rate capability and charge–discharge efficiency of batteries. Moreover, graphene can be easily combined with other materials, such as metal oxides or polymers, to create composite materials that optimize battery performance. For instance, graphene oxide (GO) can be reduced to form conductive graphene sheets that are ideal for fast charge and discharge cycles.

Carbon nanotubes (CNTs), cylindrical nanostructures formed by rolling graphene sheets, provide distinct advantages as conductive additives and structural reinforcements in battery electrodes. Their one-dimensional geometry offers exceptional electrical conductivity, mechanical strength, and flexibility, all of which contribute to enhancing battery performance and durability. When incorporated into electrode materials, CNTs form a conductive network that supports efficient ion and electron transport throughout the composite. Additionally, functionalization of CNTs can improve their compatibility with battery components—such as lithium or sodium salts—and increase the overall electrochemical stability of the system.

Carbon nanotubes (CNTs) are cylindrical nanostructures formed by rolling single graphene sheets into seamless tubes with diameters near one nanometer. Despite their nanoscale size, CNTs exhibit exceptional mechanical strength—approximately 100 times that of steel—and electrical conductivity comparable to copper. These properties make CNTs highly advantageous as conductive additives in battery electrodes.

In cathode applications, CNTs dramatically reduce the required amount of conductive additive to about one-fifth compared to conventional carbon black, due to their superior conductivity and high aspect ratio. This reduction frees volume for active materials, thereby enhancing energy density and cost efficiency.

In anodes, CNTs play a crucial role when combined with silicon-based materials, which suffer from substantial volume changes during charge–discharge cycles, leading to mechanical degradation. CNTs form flexible, robust conductive networks that accommodate silicon’s expansion and contraction, improving mechanical stability and electrode longevity. This also enables thinner electrodes with faster charging capabilities and extended cycle life.

**Figure 7:**
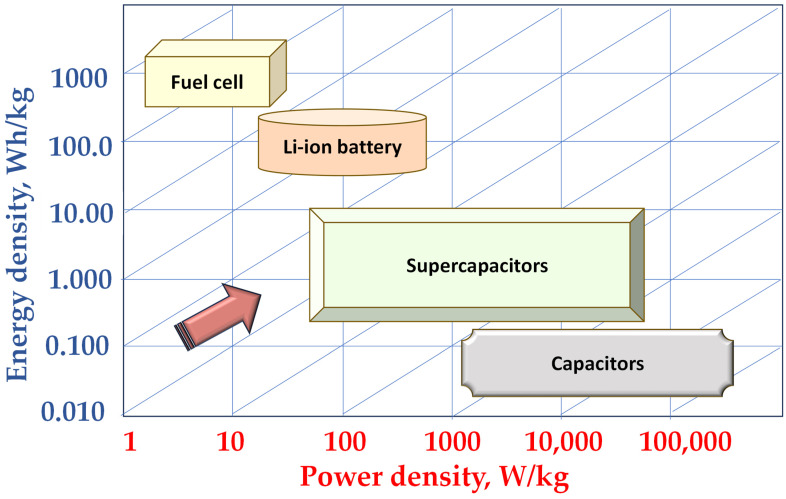
Ragonne plot for different energy storage solutions, scaled for comparison in their power density and energy density [94].

Beyond standalone CNTs, nanocomposites that integrate CNTs and graphene with metal nanoparticles, metal oxides (e.g., MnO_2_, Co_3_O_4_), polymers, or ceramics show promising synergistic effects. These composites enhance electrochemical stability, capacity, and cycle life by addressing challenges such as volume expansion and structural degradation. Fine-tuning the composition and morphology of these nanocomposites allows for tailored electrode properties, positioning them as key components in next-generation energy storage devices. Figure 7 presents the Ragone plot for fuel cells, Li-ion batteries, cupercapacitors and capacitors, scaled for comparison in their power density and energy density. A Ragone plot is a graphical tool used to compare the energy density and power density of different energy storage devices such as batteries, supercapacitors, and fuel cells. The plot typically displays power density on the x-axis (in watts per kilogram) and energy density on the y-axis (in watt-hours per kilogram), allowing a clear visual comparison of performance. Devices located toward the top-right corner of the plot are considered ideal, as they offer both high energy storage capacity and rapid energy delivery.

The integration of graphene, CNTs, and nanocomposites in battery technology has led to notable improvements in the energy density, power density, and cycle life of batteries. These materials continue to be at the forefront of research aimed at developing high-performance, long-lasting, and energy-efficient batteries for a wide range of applications, from portable electronics to electric vehicles and grid storage solutions. Their ability to enhance conductivity, reduce degradation, and increase storage capacity makes them key components in the ongoing advancement of battery technology.

There are many excellent reviews and research papers on the topic of Nanofiber-Based Innovations in Energy Storage Systems, and the field of research and interest is growing rapidly [95,96,97,98,99,100,101,102,103,104]. This review paper distinguishes itself from many previous reviews on nanofibers in energy storage by integrating a novel and forward-looking perspective on genetically modified nanofibers, an area that has been underexplored in the earlier literature. While past reviews have extensively covered the roles of conventional nanofibers—emphasizing properties like high surface area, porosity, and tunable morphology—this paper introduces bioengineered fibers as a transformative development. These genetically modified nanostructures are highlighted for their customizability at the molecular level, opening new pathways for tailoring ion transport, electrode architecture, and device performance.

One of the major highlights of this paper is its focus on the interface between biotechnology and materials science. It explores how genetic and molecular engineering of fibrous materials enables not only improved electrochemical properties but also the integration of sustainable, biocompatible, and potentially self-healing components into energy storage devices. This approach is especially relevant for next-generation systems, such as flexible electronics and environmentally responsible technologies. Additionally, this review offers a critical comparison between synthetic and biopolymer-based materials, assessing both their capabilities and their limitations. It also incorporates recent innovations in polymer–nanoparticle composites, functionalized polymer matrices, and advanced polymer electrolytes, topics that are essential for pushing the boundaries of current energy storage materials.

In our view, integrating lab-on-a-chip (LOC) technology with nanofiber-based energy storage research offers a transformative approach. LOC platforms can enable rapid, high-throughput screening and precise control of nanofiber fabrication parameters, accelerating material optimization and functionalization. This miniaturized and automated testing environment could significantly reduce development time and costs, while allowing a detailed study of ion transport, mechanical resilience, and electrochemical behavior at the microscale. We believe that leveraging LOC systems will be crucial for translating genetically engineered nanofiber innovations from lab-scale proof-of-concept to scalable, commercially viable energy storage devices.

In contrast to previous reviews, which often focused on performance metrics or specific types of batteries (e.g., lithium-ion), this paper takes a broader yet more integrative view, connecting materials design with sustainability and scalability challenges. Its conclusions guide future research toward enhancing conductivity, manufacturing feasibility, and reducing environmental impact, all of which are essential for the practical deployment of nanofiber-based systems in commercial energy storage.

## 5. Conclusions

Energy storage technologies—batteries, supercapacitors, and fuel cells—are vital for efficient energy management and renewable integration, each with unique strengths and limitations. Advances in genetically modified nanofibers and biopolymers are transforming this field by offering sustainable, high-performance materials with improved conductivity, mechanical properties, and biodegradability. These bioengineered polymers present promising alternatives to traditional synthetics, addressing environmental concerns while enhancing energy storage capabilities. However, challenges in large-scale production, long-term stability, and regulatory frameworks remain.

Continued interdisciplinary research, including synthetic biology, AI-driven design, and microfluidics-based LOC integration, is essential to fully realize the potential of genetically modified nanofibers in next-generation energy systems.

Building on the promising advances highlighted in this review, future research in nanofiber-based energy storage should focus on several critical directions. Firstly, the integration of genetically modified nanofibers into commercial-scale manufacturing must be prioritized, including the development of cost-effective, scalable electrospinning and biofabrication techniques. Second, efforts should be made to further enhance the electrochemical properties of biopolymer-based materials through hybridization with nanomaterials, such as graphene, carbon nanotubes, or metal–organic frameworks. Third, interdisciplinary collaboration between synthetic biology, materials science, and energy engineering will be essential to design fibers with tunable functionality, improved conductivity, and long-term durability. Additionally, comprehensive in vitro and in vivo evaluations of the environmental and biological safety of these materials are necessary to ensure responsible deployment. Finally, the development of fully biodegradable, self-healing, and flexible nanofiber systems could revolutionize wearable and implantable energy devices, aligning with the global shift toward more sustainable and human-centric energy technologies.

## Figures and Tables

**Table 1:** Synthetic and biopolymer-based materials.

Feature	Synthetic Polymers	Biopolymers
Conductivity	Higher, but often requires doping	Lower, needs modification
Stability	Good thermal and chemical stability	Prone to degradation
Sustainability	Fossil fuel-derived, non-biodegradable	Renewable and biodegradable
Cost	Often cheaper and mass-produced	Higher cost, but improving

**Table 2:** Patents related to spider genes and their applications.

Patent	License Holder	Approved	Silk Gene (Spider Species)	Application/Use
US 5989894	University of Wyoming	Yes	*Nephila clavipes*	Production of silk threads
WO 9949035	Zeneca	No	*Segestria florentina*	Insecticide for GM crops
US 5763568	Zeneca	Yes	*Hadronyche of Atrax*	Insecticide for GM crops
US 5658563	FMC Corp and NPS	Yes	*Diguetia canities*	Insecticide for GM crops
US 5688764	NPS	Yes	*Calisoga*	Insecticide for GM crops
US 5441934	FMC Corp and NPS	Yes	*Tegenaria*	Insecticide for GM crops
US 5457178	FMC Corp and NPS	Yes	*Filistata hibernalis*	Insecticide for GM crops
GB 2288807	British Biotechnology	Yes	*Latrodectus mactans* (Black Widow)	Insecticide for GM crops
WO 1255195	FMC Corp	No	*Diguetia canities*	Insecticide for GM crops
WO 9116351	U.S. Secretary of the Army	No	*Nephila clavipes*	New textile fibers

**Table 3:** Overall application of nanofibers in energy storage, and their comparison.

**Application**	**Performance**	**Impact**	**References**
Li-ion battery	Enhancing ion transport and energy storage capacity	Increase in battery capacity and safety	[60,68,72,74]
Supercapacitors	Increase in surface area for discharge cycles High energy density capacitors	High energy density Faster energy storage	[66,69,76,78]
Composite materials for energy storage	Combination with conducive polymers for electrical conductivity	Reinforcement of durability Better structural integrity	[82,85,86,87,88,89]
Flexible and wearable energy storage	Wearable supercapacitors energy storage	Wearable electronics	[90,91,92,93]

## Abbreviations

The following abbreviations are used in this manuscript:

CNFs	Carbon nanofibers
GAs	Graphene aerogels
GM	Genetically modified
GO	Graphene oxide
LIBs	Lithium-ion (Li-ion) batteries
PAN	Polyacrylonitrile
PE	Polyethylene
PEO	Polyethylene oxide
PP	Polypropylene
PVDF	Polyvinylidene fluoride
